# EFFECTS OF ENGAGEMENT IN PHYSICAL ACTIVITY BY INFORMAL CAREGIVERS ON THEIR QUALITY OF LIFE

**DOI:** 10.1093/geroni/igad104.1385

**Published:** 2023-12-21

**Authors:** Shamirah A’Azman, Pildoo Sung, Rahul Malhotra

**Affiliations:** Duke-NUS Medical School, Singapore, Singapore; Hong Kong Baptist University, Hong Kong, Hong Kong; Duke-NUS Medical School, Singapore, Singapore

## Abstract

Informal caregivers are more likely to have poorer health and wellbeing than non-caregivers. Studies have shown the health benefits of engagement in physical activity (PA) among caregivers. However, little is known about whether and how caregiver engagement in PA promotes or protects caregiver wellbeing. We examined (1) the association of caregiver engagement in PA with caregiver quality of life (QoL) and (2) the moderating effect of caregiver engagement in PA on the relationship between caregiving stressors and QoL. We used data on 278 care-recipient/caregiver dyads from the baseline interview of a longitudinal study of caregivers of older adults in Singapore. Multivariable regression first examined whether caregiver engagement in PA (assessed by number of days in a week with at least 30 minutes of PA, self-reported) was associated with physical, psychological, social, and environmental domains of QoL. Subsequently, interaction terms between caregiving stressors (care-recipient physical, memory, behavioral, and mood impairments) and caregiver engagement in PA were introduced and tested. We found that caregiver engagement in PA had a positive association with psychological, social, and environmental domains of QoL. Furthermore, caregiver engagement in PA mitigated the negative association between care-recipient mood impairment and caregiver QoL in the physical and social domains. Our findings suggest that QoL of informal caregivers of older adults may be improved or protected by their regular engagement in PA, especially if their care-recipients have mood impairments. Providers serving caregivers should encourage caregivers to engage in PA and equip them with the necessary support to do so.

